# m6A mRNA methylation-mediated MAPK signaling modulates the nasal mucosa inflammatory response in allergic rhinitis

**DOI:** 10.3389/fimmu.2024.1344995

**Published:** 2024-07-01

**Authors:** Ruikun Wang, Jieqiong Liang, Qian Wang, Yiming Zhang, Yingxia Lu, Xiaojun Zhan, Shan Wang, Qinglong Gu

**Affiliations:** ^1^ Department of Otorhinolaryngology Head and Neck Surgery, Children’s Hospital, Capital Institute of Pediatrics, Beijing, China; ^2^ Capital Institute of Pediatrics, Peking University Teaching Hospital, Beijing, China; ^3^ Graduate School of Peking Union Medical College, Capital Institute of Pediatrics, Beijing, China; ^4^ Beijing Municipal Key Laboratory of Child Development and Nutriomics, Capital Institute of Pediatrics, Beijing, China

**Keywords:** m6A, allergic rhinitis, ALKBH5, MAPK pathway, inflammation

## Abstract

**Background:**

Allergic rhinitis (AR) is a complex disease in which gene-environment interactions contribute to its pathogenesis. Epigenetic modifications, such as N6-methyladenosine (m6A) modification of mRNA, play important roles in regulating gene expression in multiple physiological and pathological processes. However, the function of m6A modification in AR and the inflammatory response is poorly understood.

**Methods:**

We used the ovalbumin (OVA) and aluminum hydroxide to induce an AR mouse model. Nasal symptoms, histopathology, and serum cytokines were examined. We performed combined m6A and RNA sequencing to analyze changes in m6A modification profiles. Reverse transcription-quantitative polymerase chain reaction (RT-qPCR) and methylated RNA immunoprecipitation sequencing qPCR (MeRIP-qPCR) were used to verify differential methylation of mRNAs and the m6A methylation level. Knockdown or inhibition of *Alkbh5* in nasal mucosa of mice was mediated by lentiviral infection or IOX1 treatment.

**Results:**

We showed that m6A was enriched in a group of genes involved in MAPK signaling pathway. Moreover, we identified a MAPK pathway involving *Map3k8, Erk2, and Nfκb1* that may play a role in the disrupted inflammatory response associated with nasal inflammation. The m6A eraser, *Alkbh5*, was highly expressed in the nasal mucosa of AR model mice. Furthermore, knockdown of *Alkbh5* expression by lentiviral infection resulted in high MAPK pathway activity and a significant nasal mucosa inflammatory response. Our findings indicate that ALKBH5-mediated m6A dysregulation likely contributes to a nasal inflammatory response via the MAPK pathway.

**Conclusion:**

Together, our data show that m6A dysregulation mediated by ALKBH5, is likely to contribute to inflammation of the nasal mucosa via the MAPK signaling pathway, suggesting that ALKBH5 is a potential biomarker for AR treatment.

## 1 Introduction

Allergic rhinitis (AR) is a global health problem with increasing prevalence (1–3). AR impairs the quality of social, school and work life, and is associated with substantial economic costs (4, 5). AR is an allergic airway disease resulting from immunoglobulin E (IgE)-mediated mucosal inflammatory responses to inhaled allergens (6, 7). The pathological mechanism of AR is complicated and is associated with genetic and environmental factors. Environmental determinants, such as allergen exposure, air pollution, climate change, and viral infection may underlie much of the increase in AR prevalence (8–11). In addition, specific epigenetic changes may contribute to the development of AR. Recent studies have shown allergic children to have altered DNA methylation at CpG sites in airway epithelial cells and hypermethylation of DNA can lead to decreased expression of IFN-γ in patients with AR (12, 13). Some findings indicate that an increase in histone deacetylase activity might contribute to the pathogenesis of AR by increasing pro-inflammatory cytokines and decreasing anti-inflammatory cytokines (14–17). Other studies have shown that miRNA-mediated regulation of gene expression plays an immune-modulating role in AR. High levels of miR-221 and miR-142–3p expression promote mast cell degranulation and reinforce the degranulation of mast cells in nasal mucosa, respectively, and can be biomarkers for AR (18–20). Understanding the epigenetics of AR may optimize outcomes through early diagnostics, personalized and novel therapeutics, and early prognostication.

N6-methyladenosine (m6A) methylation is a dynamic and reversible modification that is one of the most abundant modifications of eukaryotic messenger RNA (mRNA) (21, 22). It is actively involved in the critical regulation of pre-mRNA processing, miRNA processing, translation initiation, and mRNA decay (23, 24). m6A is regulated by enzymes, such as methyltransferases, demethylases, and methylated reading proteins (25–27). The methyltransferase complex is composed of methyltransferase-like 3 (METTLE3), methyltransferase-like 14 (METTL14) and wilms tumor 1-associated protein (WTAP), which are collectively referred to as m6A writers (28). In contrast, demethylases, such as fat mass and obesity-associated protein (FTO) (29), alkB homolog 5 (ALKBH5) (30) and ALKBH3 can act as erasers to remove m6A. m6A modification of RNAs must be recognized by m6A reader proteins (including YTH domain familie (31)) to mediate different downstream functions.

Accumulating evidence shows that m6A methylation of RNA and the regulators of this process are associated with a variety of airway diseases. mRNA levels of m6A modification enzymes, which are significantly enriched in the signaling pathways and biological processes that promote the progression of chronic obstructive pulmonary disease, are highly correlated with the occurrence of this disease (32). m6A methylation plays an essential role of in the pathogenesis of asthma; mRNA and protein levels of METTL14 and ALKBH5 are significantly decreased in asthma (33). In addition, differentially methylated mRNAs in lung tissues of asthmatic mice are involved in several immune function-relevant signaling pathways (34). Severe acute respiratory syndrome coronavirus 2 genomic RNA, as well as the negative-sense RNA, are dynamically m6A-modified in mammal cells (35). Moreover, numerous studies have shown that m6A regulates various immune responses and inflammation processes. METTL3-mediated m6A methylation of mRNA promotes dendritic cell activation and function and strengthens TLR4/NF-κB signaling-induced cytokine production (36). m6A modification is an important factor in the regulation of M1 and M2 polarization of macrophages (37–39) and plays an important role in the realization of macrophage functions (40). Other results indicate that YTHDF2 knockdown activates MAPK and NF-κB signaling pathways, which promote the expression of proinflammatory cytokines and aggravate the inflammatory response (41).

**Figure 1:**
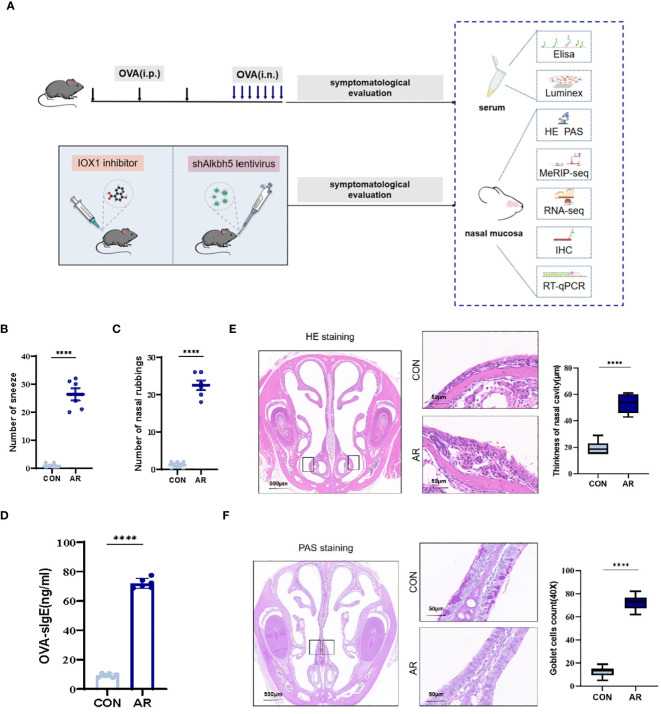
Increase of airway inflammation in OVA-induced AR. **(A)** The flowchart of this study. **(B)** The levels of sneezing **(C)** and nose scratching(n=6). **(D)** OVA-slgE levels in the serum(n=6). **(E)** HE staining for analysis of thickness of nasal cavity(n=6). **(F)** PAS staining for analysis of goblet cells(n=6). Data are expressed as mean ± SEMs. ****p<0.0001. OVA, ovalbumin; i.p., intraperitoneal injection; i.n., intranasal injection.

As an allergen-mediated disorder of the nasal passage, the pathophysiology of AR involves both an immune response and an inflammatory process. It also shares several similarities with asthma, which is another allergic disease of the lower respiratory tract. However, whether m6A methylation is involved in the pathophysiology of AR has not been elucidated. In the present study, we first investigated the dynamic changes of transcriptome-wide m6A methylation levels in the nasal mucosa of AR model mice and determined the expression of the major m6A regulator, ALKBH5 (**Figure 1A**). m6A was enriched in several inflammatory response and immune function-relevant signaling pathways. We identified a MAPK pathway involving *Map3k8, Erk2, and Nfκb1*, which may play a role in the dysfunctional inflammatory response associated with nasal inflammation. In addition, we found that *Alkbh5* knockdown or inhibitor treatment protects against ovalbumin (OVA)-induced AR. Mechanistically, ALKBH5 regulates the immune response to AR through the MAPK/ERK pathway. Expression of genes associated with the MAPK/ERK pathway was altered as a result of *Alkbh5* knockdown. This study contributes to our understanding of the role of m6A modification in the immune response to AR.

## 2 Materials and methods

### 2.1 Animals

Male wild-type C57BL/6 mice, aged 6–8weeks, were used in this investigation. They were all obtained from SPF (Beijing) Biotechnology Co., Ltd. The Ethics Committee of Capital Institute (Beijing, China) gave its approval to the protocols for animal studies, with Ethic Certificate No: DWLL2023013. Guiding the mice through their care, housing, and ultimate demise, the National Institute of Health Guide for the Care and Use of Laboratory Animals provided guidance.

The animal model of AR was constructed as described previously (42). The ovalbumin (OVA) and aluminum hydroxide were used to induce the AR in mice. In conclusion, 50 μg of OVA (Sigma-Aldrich, St. Louis, MO, USA) was dissolved using saline. Intraperitoneally administering a mixture of 1 mg of aluminum hydroxide (Thermo Fisher, CA, USA) and OVA to mice over the course of 1, 7, and 14 days (sensitization stage) was done. The mice were then subjected to an intranasal instillation of 20 μL of 10% OVA solution in each nostril.

### 2.2 Evaluation of nasal symptoms

The number of nasal rubbing and sneezing behaviors were evaluated. Following the final OVA intranasal challenge in day 28, the nasal symptoms were recorded for ten minutes by two separate researchers.

### 2.3 OVA-specific IgE, cytokine and chemokine analyses

Blood samples are centrifuged to get serum samples. Six serum samples in each group were analyzed for OVA-specific IgE levels using ELISA kits (Mlbio, Shanghai, China). Using the Bio-Plex Pro Mouse Cytokine 23-plex Assay (Bio-Rad Laboratories), we measured 23 cytokines, chemokines, and growth factors in the three serum samples in each group following the manufacturer’s instructions.

### 2.4 Histopathological analyses of nasal tissues

Formaldehyde solution of 10% neutral buffered nature was employed to secure the heads of mice (Sigma-Aldrich, St.) for a week. And the heads of mice were decalcified in 0.1 M EDTA buffer (Bio-solution, Suwon, Korea) and then embedded in paraffin for two weeks. Hematoxylin (Sigma-Aldrich, St.) and eosin (Sigma-Aldrich, St. Louis,MO, USA) was employed to stain the 5 μm slices of blocks. The remaining slides were stained with Periodic acid-Schiff (PAS, Sigma-Aldrich, St. Louis, MO, USA) for analyzing goblet cell hyperplasia. Slide digital scanners from 3DHISTECH (Budapest, Hungary) were utilized to scan the slides, which were then read with Pannoramic case software (3DHISTECH, Budapest, Hungary). Six nasal mucosa tissues in each group were used for analyses.

### 2.5 Immunohistochemistry analysis

Immunohistochemistry (IHC) was performed as described earlier. After dewaxing and hydration, antigens were extracted from nasal mucosa and heated in citrate buffer (Sigma-Aldrich, PBS1). After 15 minutes of treatment with 3% hydrogen peroxide (ZSGB-BIO ORIGENE, SP-9000) to inhibit endogenous peroxidase activity, the slides were incubated with 5% goat serum at room temperature for 30 minutes (Bioss Biotechnology Company, C-0005). Next, the tissues were probed with primary antibody against m6A(CST,1:100), ALKBH5 (Abcam, 1:400), FTO(CST,1:200), METTL3(CST,1:200),METTL14(CST,1:200) overnight at 4°C. For negative controls, irrelevant primary antibodies were applied. Incubating the secondary antibodies with the sections at room temperature for 1 hour, and they were bound to horseradish peroxidase (SP-9000, ZSGB-BIO ORIGENE). The slices were washed with phosphate-buffered saline (PBS), incubated in a solution containing horseradish enzyme labeled avidin chain for 30 minutes, 37°C, and then rinsed again.

### 2.6 Reverse transcription and quantitative real-time polymerase chain reaction

Under the manufacturer’s protocol, Trizol reagent (Invitrogen, CA, United States) was employed to extract total RNAs from the nasal mucosa. A single sample was composed of five nasal mucosa tissue from five mice, and three technical replications were conducted in each experiment. NanoDrop ND-1000 instrument was utilized to evaluate the purity and amount of RNA specimens. Using the Revert Aid First Strand cDNA Synthesis Kit (ABM,Richmond, Canada), a Reverse Transcripting Ablation Method was employed to prepare cDNA. Real-time polymerase chain reaction (RT-qPCR) was employed to assess the cDNA, with the Maxima SYBR Green/ROX qPCR Master Mix (ABM) being employed to determine the RNA concentrations of the targeted genes. The RT-qPCR procedure was conducted with GAPDH as an internal control, with one cycle of 95°C for 10 minutes, 35 cycles of 95°C for 35 seconds, and 60 cycles of 60°C for 60 seconds.

### 2.7 Western blot

As with RT-qPCR, a single sample was composed of five nasal mucosa tissue, and three technical replications were conducted in each experiment. Nasal mucosa samples were extracted with RIPA lysis buffer (Beyotime, Shanghai, China) and centrifuged at 4°C and14,000 rpm for 15 min. Protein samples were separated via 7.5% (wt/vol) SDS-PAGE and transferred onto PVDF membranes (Millipore, Billerica, MA, USA). The membranes were blocked with 5% skim milk for 1.5 h at room temperature. Then, the primary antibodies were incubated overnight at 4°C. The membranes were washed three times and incubated with secondary antibodies for 1 h at room temperature. The primary antibodies used in this study were mouse anti-ALKBH5 monoclonal antibody (Abcam,1:1000), GAPDH(Abcam,1:1000), NF-κB p65(CST,1:500) and Phospho-NF-κB p65(CST,1:500). The secondary antibodies used was anti-rabbit HRP-conjugated antibody (CST; 1:5000). Then, protein bands were developed with ECL reagents, and images were acquired with the Bio-Rad Imaging system. In order to avoid affecting the accuracy of quantification due to inconsistent operating conditions, the reference and target proteins were on the same imprinted membrane in this study. We used Western Blot Stripping Buffer (Abcam) uniformly to remove antibodies from Western blots so that the blots may be reprobed with different antibodies. Stripping and reprobing a western blot is a method in which the primary and secondary antibodies are removed from a western blot so the blot can be reprobed.

### 2.8 ALKBH5 inhibitor

IOX1 was found to be an inhibitor of ALKBH5 in previous studies (43–45), which inhibited ALKBH5 activity in a cofactor 2-oxoglutarate oxygenase competitive manner. According to the IOX1(MCE, New Jersey, USA) product instructions, the working solution for *in vivo* experiments was prepared on the same day and used on the same day. The stock solution was prepared using DMSO(MCE, New Jersey, USA). Using 1 mL working solution as an example, 100 μL of 20.8 mg/mL clarified DMSO stock solution was added to 900 μL of 20% aqueous SBE-β-CD saline solution and mixed evenly. Preparation of 20% SBE-β-CD in Saline (4°C, 1 week storage): 2 g SBE-β-CD (sulfobutyl ether β-cyclodextrin) powder was fixed in 10 mL of normal saline and completely dissolved until clear. For IOX1 treatment, mice were intra-peritoneally injected with 10 mg/kg/day from day 21 to 28.

### 2.9 Plasmid construction

This study used the lentiviral knockdown system composed of pLV3-CMV-GFP-Puro, PG-P1-VSVG, PG-P2-REV, and PG-P3-RRE. In the LV3 vector, mouse ALKBH5 shRNA was inserted between the HIV 3 LTR and the H1 promoter. Transducing 293T cells was used to titrate the lentivirus (ten-fold serial dilution). The lentivirus utilized in this investigation typically had a titer between 1,108 and 11,010 TU/ml. In H293T cells, the effectiveness of the knockdown was assessed by RT-qPCR. The following oligonucleotide sequences were used for ALKBH5 shRNA: mouse ALKBH5-shRNA: 5′-CTGCGCAACAAGTACTTCTTC-3′; and scramble control:5′-TTCTCCGAACGTGTCACGT-3′.

### 2.10 Lentivirus intranasal insillation

The intranasal insillation of lentivirus was performed according to previous report (46). 20μL lentivirus (2 × 10^6^ IFU) konckout ALKBH5 was intranasally administered 1 h before the nasal infusion of AR+LV-sh*Alkbh5* group mice with OVA from day 21 to 28. The AR+LV-NC group was treated with 2 × 10^6^ IFUs of an empty lentivirus vector, whereas the control (CON) group was treated with isopyknic PBS.

### 2.11 MeRIP-seq and RNA-seq

A single sample was composed of ten nasal mucosa tissues from mice, and three technology replications were conducted. Using TRIzol reagent, a total RNA was quickly isolated and quantified. Two parts of the RNA were split; one was kept as an input, and the other was employed to enrich m6A-methylated RNA fragments by immunoprecipitation with a m6A-specific antibody (Synaptic Systems, Germany). Employing the Ribo-Zero rRNA Removal Kit (Illumina, San Diego, CA, USA), 1µg of rRNAs were eliminated for RNA-seq, while the Tru Seq Stranded Total RNA Library Prep Kit (Illumina, San Diego, CA) was utilized. Utilizing Agilent Technologies’ Bio Analyzer 2100 system (USA), RNA-seq libraries were constructed and then regulated and assessed.

The Bio Analyzer 2100 system (Agilent Technologies, USA) was employed to regulate and assess both libraries afterwards. The 10 pM libraries were denatured as single-stranded DNA molecules and amplified *in situ* as clusters. MeRIP-seq and RNA-seq procedures were carried out on an Illumina NovaSeq 6000 instrument (CHI BIOTECH CO.,LTD, Shenzhen, China) with150-bp paired-end reads, following the manufacturer’s protocol.

### 2.12 MeRIP-qPCR

MeRIP-qPCR was conducted according to a protocol slightly modified based on the previously described (47). Three differentially methylated RNA sites were selected to design specific primers for MeRIP-qPCR using NCBI Primer-Blast. Sangon Biotech Co. produced the forward and reverse primers (Shanghai, China). The RNA was used for m6A immunoprecipitation with m6A antibody (Synaptic Systems, USA) in immunoprecipitation (IP) buffer to obtain m6A pulldown components (m6A IP portion). RNA from immunoprecipitated m6A was immunoprecipitated using Dynabeads Protein A (Thermo Fisher Scientific, USA), followed by elution. The RNA concentration of m6A IP RNAs recovered by ethanol precipitation was determined by spectrophotometry (Thermo Fisher Scientific, USA). Following that, the input and m6A IP RNA were subjected to RT-qPCR on QuantStudio™ 7 System, as described above.

### 2.13 Sequencing data analysis

The FASTP software was used to remove the low-quality reads and trim adaptor of the IP and input samples (48). Sequence quality of IP and input samples was examined by FastQC (https://www.bioinformatics.babraham.ac.uk/projects/fastqc/) (49) and RseQC (http://rseqc.sourceforge.net/) (50). Clean reads from all libraries were then mapped to the reference genome Homo sapiens via HISAT2 (V2.1.0) (51), after 3′ adaptor-trimming and low-quality read removal via Cutadapt software (v1.9.3) (52). m6A peak calling and analysis of differentially methylated peaks were performed by R package exomePeak2 (53). The MEME (http://meme-suite.org) (54) and HOMER (http://homer.ucsd.edu/homer/motif) were used to search for motifs enriched in m6A peaks of each group of samples. The peak map was visualized by IGV software (v2.3.5). Heatmaps of the *p*-values of motifs were generated using the pheatmap R package. In order to view m6A peaks throughout the whole transcript, Integrative Genomics Viewer was employed. The R package edgeR (55) was used to identify differentially expressed transcripts and genes, and the threshold was set to |log2fold change (FC)|≥1 and *p* value < 0.05. Gene Ontology (GO) enrichment analysis and Kyoto Encyclopedia of Genes and Genomes (KEGG) pathways analyses were conducted on differentially methylated and differentially expressed mRNAs using ClusterProfiler (ver. 4.2.2). The ggplot2 R package was used to create plots of GO/KEGG terms.

### 2.14 Quantification and statistical analysis

Data from three experiments, conducted independently, was expressed in mean ± SEM. Student’s t-test and one-way ANOVA were employed to contrast the statistical significance between the two and more than two groups, and GraphPad Prism 5.0 was utilized for statistical analysis. *P* values of less than 0.05 were presented as either *p<0.05, **p<0.01, *** *p* < 0.001 or ****p<0.0001.

## 3 Results

### 3.1 Establishment of AR mice

We established an AR model as previously described. Nasal rubbing and sneezing behaviors are the primary symptoms of AR. We recorded the numbers of nasal rubbing and sneezing behaviors for ten minutes following the final nasal OVA challenge on day 28. OVA challenge caused a significant increase in sneezing (mean ± SEM: 46.50 ± 4.885 vs 4.42 ± 0.56, *p*<0.0001) and nose-scratching events (mean ± SEM: 25.42 ± 1.42 vs 2.00 ± 0.30, *p*<0.0001) compared with the CON group (**Figures 1B, C**). Furthermore, AR is characterized by the increased production of serum antigen-specific IgE. OVA-specific IgE levels were significantly higher as compared with the CON group (*p*<0.0001) (**Figure 1D**). We monitored the nasal histopathological changes in the sectioned nasal tissue of mice by HE staining. As shown in **Figure 1E**, no pathological abnormalities were observed in the nasal mucosa of the CON group. Conversely, the AR group demonstrated increased thickness (mean ± SEM: 53.17 ± 3.07 vs 19.50 ± 2.16, *p*<0.0001) because of increased accumulation of inflammatory cells under the epithelium (**Figure 1E**). Epithelial cells first encounter environmental triggers, including pathogens and allergens. Hypertrophic changes in goblet cells associated with mucous production are an important characteristic of inflammation of the respiratory epithelium. Therefore, we analyzed goblet cell hyperplasia of sectioned nasal tissue mice by PAS staining. The AR group showed significant goblet cells (mean ± SEM: 54.44 ± 2.02 vs 3.67 ± 1.56, *p*<0.0001) in nasal submucosa compared to the CON group (**Figure 1F**).

### 3.2 Overview of m6A methylation in AR nasal mucosa

**Figure 2:**
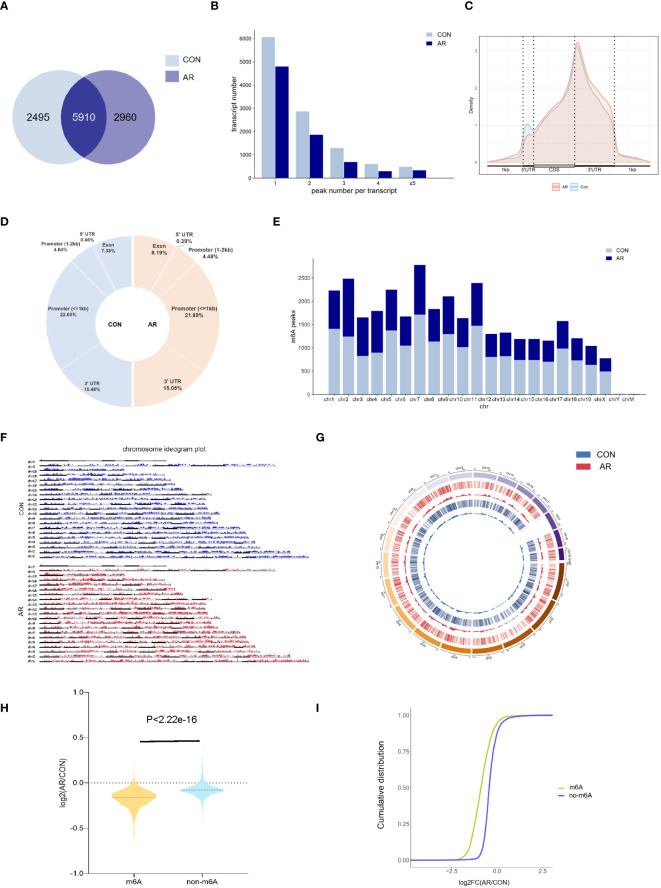
Overview of m6A transcriptome in the nasal mucosa. **(A)** The overlap of m6A-modifed sites among samples(n=3). **(B)** Numbers of transcripts containing different numbers of m6A peaks per transcript of AR and CON groups. **(C)** Priority region and average distribution of m6A peaks. **(D)** The distribution of m6A peaks among samples. **(E)** The count of m6A peaks in mouse chromosomes. **(F)** Chromosome ideogram plot of m6A peaks. **(G)** Density distribution of the m6A peaks along the chromosome. **(H)** Violin diagram and **(I)** cumulative graph of the effect of m6A methylation on gene expression.

To examine m6A regulation of altered gene expression that accompanies changes in the nasal mucosa of AR model mice, we performed methylated RNA immunoprecipitation sequencing (MeRIP-seq) on nasal mucosa tissue samples from CON group and AR group (**Supplementary Table S1**). By comparing the distributions of the MeRIP-seq reads in the nasal mucosa of mice, a total of 8,405 and 8,870 m6A peaks were identified in CON and AR groups, respectively. Of these, 5,910 peaks were common in both groups (**Figure 2A**). The 49 hyper-methylated and 3465 hypo-methylated m6A peaks within mRNAs were identified (fold changes≥2.0 and *p* < 0.05) by comparing the m6A methylation levels (**Supplementary Figure S2A**). The distribution characteristics of mRNA transcript peak frequency were analyzed in the two groups. Most transcripts contained only one m6A peak in AR and CON groups (**Figure 2B**; **Supplementary Table S2**). Metagene profile analysis indicated that m6A peaks were preferentially located in coding regions, at start codons, and near stop codons in both groups. The CON group had a higher proportion of m6A peak enrichment in 5′-untranslated regions (UTRs) than the AR group (**Figure 2C**; **Supplementary Table S2**). Furthermore, there were similar trends in the distribution patterns of the m6A peaks in genic regions for the two groups. The peaks were abundant in 3′-UTRs (30.96% in the AR group and 30.10% in the CON group) and in exon regions (14.76% and 16.39%, respectively), followed by the 5′-UTRs (0.92% and 0.70%, respectively) (**Figure 2D**; **Supplementary Table S2**). We performed motif searches for all detected peaks (**Supplementary Figure S2B**). Visualization of the distribution of the transcriptome-wide m6A peaks across the chromosomes was performed in nasal mucosa samples. We calculated the density of these differentially methylated peaks in each chromosome and found that they were not distributed homogeneously. The top three chromosomes with the largest number of peak distributions were chromosomes 7, 2, and 12 (**Figure 2E**). Besides, we mapped the differentially methylated peaks to the mouse chromosomes and visualized the peak site locations across the chromosomes using the R package RIdeogram (**Figure 2F**). The results showed that the overlapping peaks between the two groups were distributed on each chromosome, and the distribution patterns were similar (**Figure 2G**). The violin plot and cumulative density plot show the numerical distribution of gene expression after classification according to the presence or absence of m6A peak, indicated that m6A modification down-regulated gene expression (**Figures 2H, I**).

### 3.3 Enrichment clustering of m6A methylated mRNAs in AR nasal mucosa

**Figure 3:**
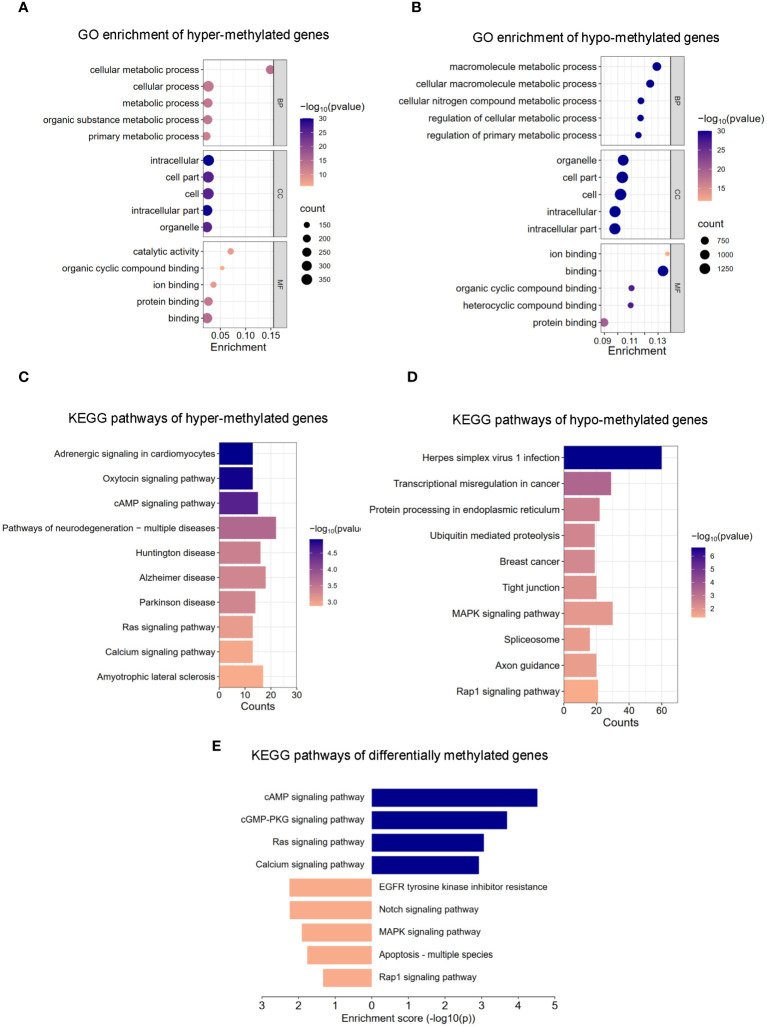
GO and KEGG pathway analysis of m6A modified transcripts. **(A)** Top 5 GO terms enriched across up-regulated m6A-tagged transcripts. **(B)** Top 5 GO terms enriched across down-regulated m6A-tagged transcripts. **(C)** Top 10 KEGG terms enriched across up-regulated m6A-tagged transcripts. **(D)** Top 10 KEGG terms enriched across down-regulated m6A-tagged transcripts. **(E)** KEGG terms related to immune response and inflammation reaction.

The m6A methylation levels in the AR and CON groups were compared to explore the changes and functions of m6A methylation in AR. To explore the differentially methylated mRNAs and their potential roles in the pathological processes of AR, we performed Gene Ontology (GO) analyses of up- and down-methylated mRNAs (**Figures 3A, B**; **Supplementary Table S3**). For up-regulated m6A peaks, the top five most enriched biological process (BP), cellular component (CC), and molecular function (MF) are listed in **Figure 3A**. In the BP category, the main enrichment was in cellular metabolic process, cellular process, metabolic process, organic substance metabolic process, and primary metabolic process. In the CC category, the top five enriched functions were cell part, cell, intracellular. In the MF category, binding, protein binding, catalytic activity, ion binding, and organic cyclic compound binding were the most enriched terms. Whereas, GO enrichment results showed that hypo-methylated genes were significantly enriched in macromolecule metabolic process, cellular macromolecule metabolic process, cellular nitrogen compound metabolic process, regulation of cellular metabolic process,and regulation of primary metabolic process of BPs; organelle, cell part, cell, intracellular, and intracellular part of CCs; ion binding, binding, organic cyclic compound binding, heterocyclic compound binding, and protein binding of MFs (**Figure 3B**; **Supplementary Table S3**). Next, we analyzed the distributions of the differentially methylated mRNAs in KEGG categories. KEGG pathways of hyper-methylated genes were mainly enriched in “Pathways of neurodegeneration - multiple disease”, “Alzheimer disease”, “Amyotrophic lateral sclerosis”, “Huntington disease”, “cAMP signaling pathway”, “Parkinson disease”, “Adrenergic signaling in cardiomyocytes”, “Oxytocin signaling pathway”, “Ras signaling pathway” and “Calcium signaling pathway” (**Figure 3C**; **Supplementary Table S4**). KEGG pathways of hypo-methylated genes indicated significant gene enrichments in “Herpes simplex virus 1 infection”, “MAPK signaling pathway”, “Transcriptional misregulation in cancer”, “Protein processing in endoplasmic reticulum”, “Rap1 signaling pathway”, “Tight junction”, “Axon guidance”, “Ubiquitin mediated proteolysis”, “Breast cancer” and “Spliceosome” (**Figure 3D**; **Supplementary Table S4**). Moreover, **Figure 3E** shows that KEGG analysis identified several significantly enriched pathways related to immune regulation and the inflammation response, such as “MAPK signaling pathway”, “EGFR tyrosine kinase inhibitor resistance”, “Notch signaling pathway”, “PD-L1 expression and PD-1 checkpoint pathway in cancer” and “Hippo signaling pathway”. Details are presented in **Supplementary Table S5**. Taken together, these data indicate that differential m6A modification of mRNAs affects the inflammation response in AR nasal mucosa.

### 3.4 Transcriptome changes in AR nasal mucosa

**Figure 4:**
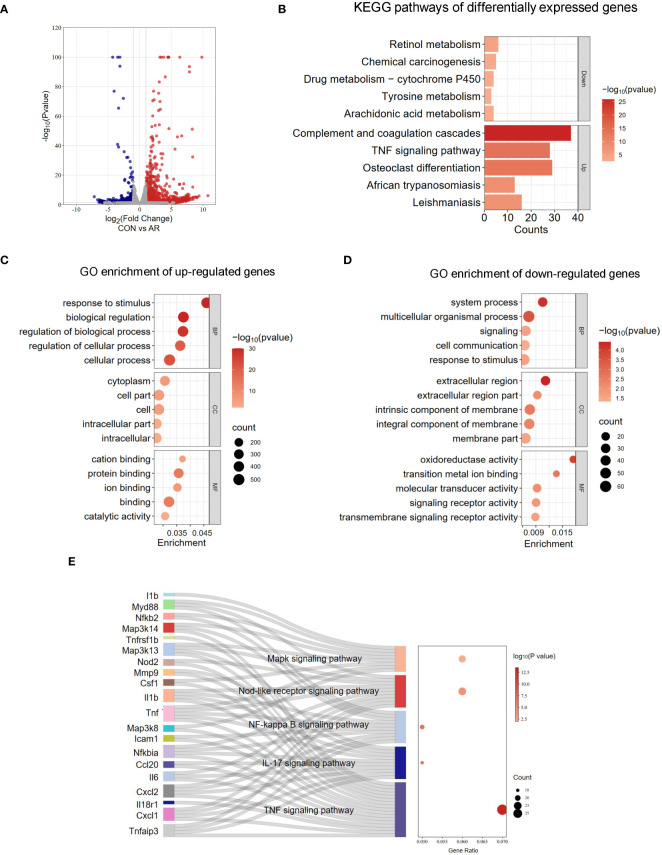
Analysis of RNA-seq. **(A)** Volcano plots revealing the expression of mRNA with statistical significance (fold changes ≥ 2.0 and *p* < 0.05). **(B)** Top 5 GO terms enriched across up-regulated mRNA transcripts. **(C)** Top 5 GO terms enriched across down-regulated mRNA transcripts. **(D)** Top 5 KEGG terms enriched across down-regulated and up-regulated mRNA transcripts. **(E)** Sankey dot pathway enrichment of regulated genes.

To evaluate the gene expression profile accompanying AR, we performed RNA-seq on mouse nasal mucosa. We identified 1,277 genes showing increased expression and 198 genes showing decreased expression (**Figure 4A**; **Supplementary Table S6**). We then conducted GO and KEGG enrichment analyses for these differentially expressed genes. In GO analysis, the up-regulated genes were mainly related to response to stimulus, regulation of biological process, and regulation of cellular process. (**Figure 4C**; **Supplementary Table S7**). The down-regulated genes were significantly associated with signaling receptor activity, transmembrane signaling receptor activity, and oxidoreductase activity (**Figure 4D**; **Supplementary Table S7**). KEGG analysis identified several significantly enriched pathways of the differentially expressed genes (**Figure 4B**; **Supplementary Table S8**). As shown in **Figure 4E**, signaling pathways enriched for the up-regulated genes included TNF signaling pathway, IL-17 signaling pathway, NF-κB signaling pathway, NOD-like receptor signaling pathway, and MAPK signaling pathway, which are related to immune regulation and the inflammation response (**Figure 4E**; **Supplementary Table S9**). These results suggested that tanscriptome changes modulates inflammation also involved in AR nasal mucosa.

### 3.5 m6A methylation increases transcription related to the MAPK/ERK pathway in AR nasal mucosa

**Figure 5:**
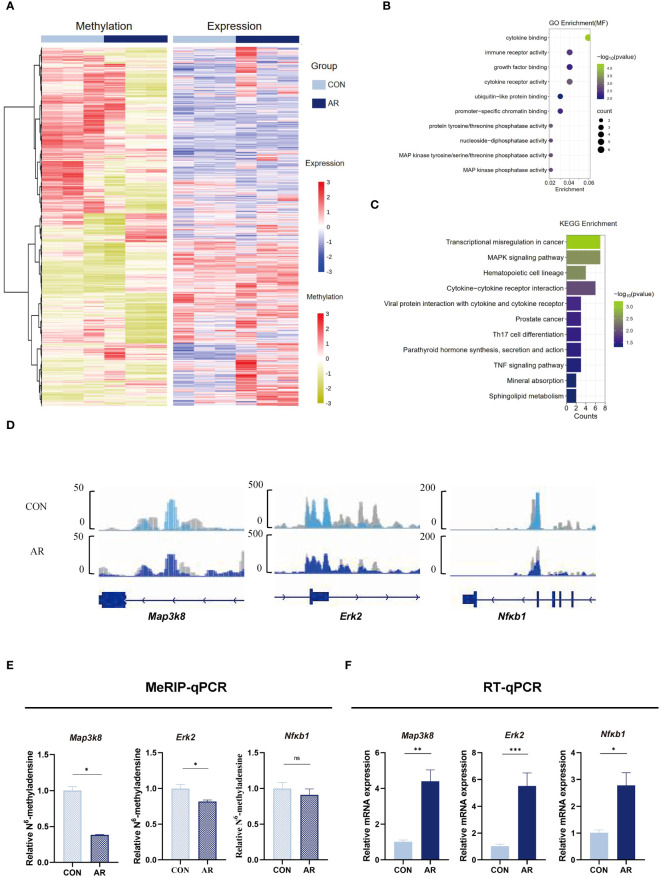
Conjoint analysis of MeRIP-seq and RNA-seq dat. **(A)** Heatmap correlating mRNA methylation levels and mRNA expression levels. **(B)** Top GO terms of differentially methylated genes that are upregulated or downregulated. **(C)** KEGG analysis of enriched pathways of differentially methylated genes that are upregulated or downregulated. **(D)** IGV plots showing methylated genes (*Map3k8, Erk2, and Nfκb1*). **(E)** MeRIP-qPCR assay indicating the m6A methylation levels of MAPK pathway-related genes *Map3k8, Erk2, and Nfκb1*(n=3). **(F)** MAPK pathway-related genes *Map3k8, Erk2, and Nfκb1* mRNA relative expression level in nasal mucosa of mice were measured by RT-qPCR(n=3). Data are expressed as mean ± SEMs. *p<0.05, **p<0.01, ****p<0.0001 or ns not significant.

We next assimilated RNA-seq data with MeRIP-seq data of promotor regions. We observed significant differential expression for 51 differentially methylated mRNA transcripts (**Supplementary Figure S4**; **Supplementary Table S10**). Given the critical function of m6A changes in controlling gene expression, we examined the relationship between m6A and mRNA levels (**Figure 5A**). To identify the roles of the differentially methylated mRNAs in the expression changes in AR, we performed GO and KEGG enrichment analyses. The GO terms most significantly enriched for hypo-methylated genes were related to immune function and inflammatory response, such as “MAP kinase tyrosine/serine/threonine phosphatase activity”, “MAP kinase phosphatase activity”, “immune receptor activity”, and “growth factor binding” (**Figure 5B**; **Supplementary Table S11**). The KEGG enrichment results showed that hypo-methylated genes with expression changes were significantly enriched in “MAPK signaling pathway”, “Cytokine-cytokine receptor interaction”, “Viral protein interaction with cytokine and cytokine receptor”, “Th17 cell differentiation”, and “TNF signaling pathway” (**Figure 5C**; **Supplementary Table S12**). MAPK can be activated by various inflammatory stimuli and plays an important role in the occurrence and development of inflammation. We visualized the distribution of the peak regions in *Map3k8, Erk2, and Nfκb1* mRNAs using IGV software, and found that the m6A methylation of these mRNAs, which are involved in the MAPK pathway, was obviously decreased in the AR group (**Figure 5D**). To determine whether m6A mediates expression of these genes, we analyzed m6A modification of *Map3k8*(Gene ID:26410), *Erk2*(Gene ID:26413) and *Nfκb1*(Gene ID:18033) transcripts by MeRIP-qPCR. The results showed decreased methylation of *Map3k8, Erk2, and Nfκb1*, although m6A modification levels of NF-κB did not differ significantly between the two groups (**Figure 5E**). This indicated decreased m6A methylation levels of these three MAPK pathway-related mRNAs in AR. RT-qPCR showed increased expression of these three genes in AR group (**Figure 5F**; **Supplementary Table S13**). Collectively, these findings indicate that m6A modification may play a regulatory role in the MAPK pathway in AR. Correlation analysis was performed between main symptoms and m6A or mRNA levels of *Map3k8, Erk2, and Nfκb1*. Number of sneeze and rubbing that considered to be specific behavioral manifestation of AR was positively correlated with m6A and mRNA levels of *Map3k8*(r=-0.951, *P*<0.001; r=-0.973, *P*<0.001; r=0.813, *P*=0.001; r=0.918), m6A and mRNA levels of *Erk2* (r=-0.794, *P*=0.002; r=-0.845, *P*==0.001; r=0.904, *P*<0.001; r=0.842, *P*=0.001), and mRNA level of *Nfκb1* (r=0.967, *P*<0.001; r=0.932, *P*<0.001). No statistically significant correlation was identified between the number of sneeze and m6A level of *Nfκb1* (*P*=0.068).

### 3.6 m6A regulator ALKBH5 is highly expressed in AR nasal mucosa

**Figure 6:**
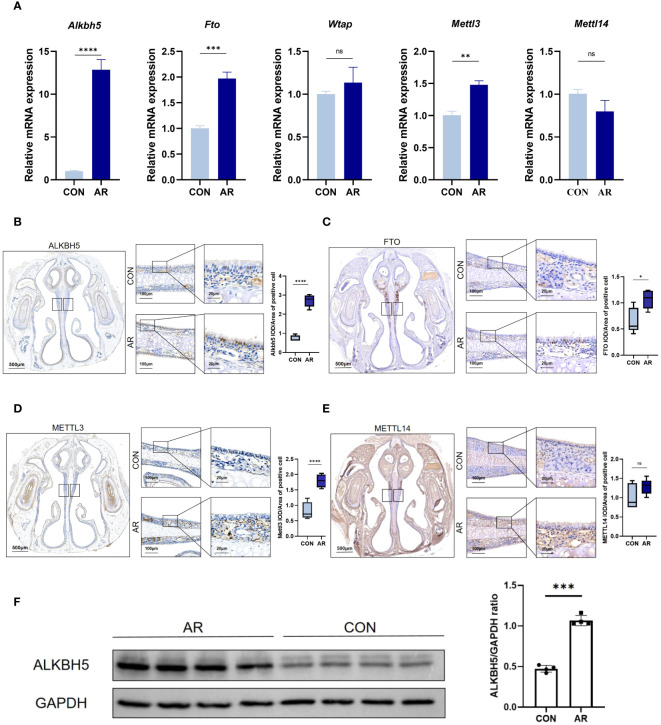
m6A modification enzymes of nasal mucosa are dysregulated in AR group. **(A)** Relative mRNA expression levels of m6A modification enzymes in nasal mucosa of mice assessed by RT-qPCR(n=3). **(B–E)** Immunostaining of mouse nasal mucosa using antibodies against the ALKBH5, FTO, METTL3, and METTL14. Quantifications of IOD/Area of positive cell are shown as box plots(n=5). **(F)** Western blot bands illustrating the protein levels of ALKBH5 and GAPDH in CON and AR groups(n=3). Data are expressed as mean ± SEMs. *p<0.05, **p<0.01, *** *p* < 0.001 or ****p<0.0001, ns not significant.

To gain insight into how the m6A methyl marks are regulated in AR nasal mucosa, we analyzed the gene expression profiles of m6A methyltransferases, demethylases, and methylated reading proteins. We also examined mRNA levels of different m6A regulator genes, including m6A writers (*Mettl3* and *Mettl14*) and two m6A erasers (*Alkbh5* and *Fto*) in ten nasal mucosa samples and matched normal tissues. AR increased the mRNA levels of m6A eraser-related genes, *Alkbh5* and *Fto* and of the m6A writer-related gene, *Mettl3*, but did not significantly change those of the m6A methylation transferases, *Wtap* and *Mettl14* (**Figure 6A**; **Supplementary Table S13**). Immunohistochemistry showed ALKBH5, FTO, and METTL3 staining to be substantially greater in mouse nasal mucosa samples compared with normal tissues (**Figures 6B–D**), while the level of METTl14 was not different (**Figure 6E**). Western blot analysis also showed that *Alkbh5* was highly expressed in nasal mucosa of AR (**Figure 6F**). Immunohistochemistry showed m6A lowly expressed in AR mice nasal mucosa samples compared with normal tissues(*P*<0.001). (**Supplementary Figure S5**). These results indicate that the regulation of m6A methytransferase and demethylase expression is related to the occurrence of OVA-induced AR in mice. To establish the association of observations with AR and m6A methylation, we performed correlation analysis between AR nasal symptoms and the levels of m6A methylation using Pearson correlation test. *P*<0.05 was considered to indicate a statistically significant difference. Firstly, there were statistically significant correlations between number of sneeze and mRNA level, protein level, positive cell of Alkbh5(r=0.970, *P*<0.001; r=0.967, *P*<0.001; r=0.945, *P*<0.001, respectively). Besides, the correlation between the number of nasal rubbing and mRNA level, protein level, positive cell of Alkbh5 was revealed to be statistically significant (r=0.979, *P*<0.001; r=0.977, *P*<0.001; r=0.955, *P*<0.001, respectively). Correlation analysis between the number of nasal rubbing, sneeze and m6A expression level showed a statistically significant difference (r= -0.737, *P*=0.037, r=-0.775, *P*=0.024). Consistent results were obtained by correlation analysis between OVA-sIgE level and the levels of m6A methylation (**Supplementary Table S15**).

### 3.7 *Alkbh5* knockdown in the nasal mucosa relieves AR allergy symptoms

**Figure 7:**
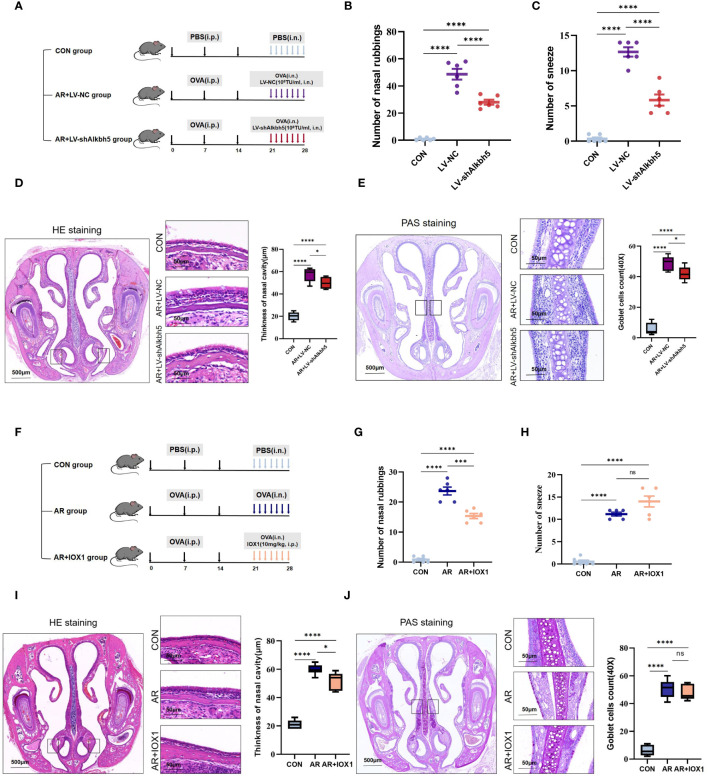
*Alkbh5* knockdown in the nasal mucosa relieves AR allergy symptoms. **(A)** Schematic illustrating CON group, AR+LV-NC group and AR+LVsh*Alkbh5* group. **(B, C)** The levels of sneezing and nose scratching in each group(n=6). **(D)** HE staining for analysis of thickness of nasal cavity(n=3). **(E)** PAS staining for analysis of goblet cells(n=3). **(F)** Schematic illustrating CON group, AR group and AR+IOX1 group. **(G, H)** The levels of sneezing and nose scratching in each group(n=6). **(I)** HE staining for analysis of thickness of nasal cavity(n=3). **(J)** PAS staining for analysis of goblet cells (n=3). Data are expressed as mean ± SEMs. *p*<0.05, *** *p* < 0.001, ****p<0.0001 or ns not significant.

Our data collectively showed that the expression levels of common m6A methyltransferases and demethylases, especially ALKBH5, significantly changed in the nasal mucosa of AR model mice, indicating that m6A modification may play an important role in this process. To investigate AR further, we altered the m6A levels in the mouse nasal mucosa by knocking down *Alkbh5* using a lentivirus plasmid (**Supplementary Figure S6A**). RT-qPCR showed efficiency of *Alkbh5* knockdown (**Supplementary Figure S6B**). To further investigate the function of ALKBH5 in AR, we examined the effects of ALKBH5 downregulation on AR. Lentivirus was administered to nasal mucosa in nasal drops (**Figure 7A**). We found that ALKBH5 was down-regulated in nasal mucosa tissues after administered(**Supplementary Figures S6C, D**). At least 7 days later we examined the primary AR symptoms of nasal rubbing and sneezing behavior. We recorded the number of nasal rubbing and sneezing behaviors in CON, AR+LV and AR+LV-sh*Alkbh5* groups (**Figures 7B, C**). Lentivirus challenge caused a significant decrease in sneezing (mean ± SEM: 5.83 ± 0.79 vs 12.67 ± 0.67, *p*<0.0001) and nose-scratching events (mean ± SEM: 28.00 ± 1.83 vs 48.67 ± 3.92, *p*<0.0001) compared with the AR+LV-NC group. The AR+LV-sh*Alkbh5* group showed decreased width of the nasal cavity (mean ± SEM: 49.83 ± 2.12 vs 57.33 ± 2.51, *p* = 0.049) and decreased goblet cell number in nasal submucosa (mean ± SEM: 42.00 ± 1.85 vs 19.00 ± 1.92, *p* = 0.025) compared with the AR+LV-NC group (**Figures 7D, E**). Next, we chose 10 mg/kg as the most appropriate IOX1 concentration (**Figure 7F**). IOX1 administration also effectively decreased the nose-scratching events (mean ± SEM: 15.33 ± 0.843 vs 12.67 ± 0.67, *p*<0.0001) (**Figure 7G**), nasal cavity width (mean ± SEM: 52.00 ± 2.50 vs 59.83 ± 1.49, *P* = 0.023) (**Figure 7I**) and goblet cell number in the nasal submucosa (mean ± SEM: 47.67 ± 2.26 vs 50.67 ± 2.70, *p*=0.023) (**Figure 7J**) compared with the AR group. However, the number of sneezes (mean ± SEM: 14.00 ± 1.21 vs 11.17 ± 0.67, *p* = 0.051) was not significantly different between AR and AR+IOX1 groups (**Figure 7H**). Taken together, the above data suggested that *Alkbh5* knockdown in the nasal mucosa relieves AR allergy symptoms.

### 3.8 *Alkbh5* knockdown in the nasal mucosa attenuates MAPK pathway upregulation and decreases the inflammatory response

**Figure 8:**
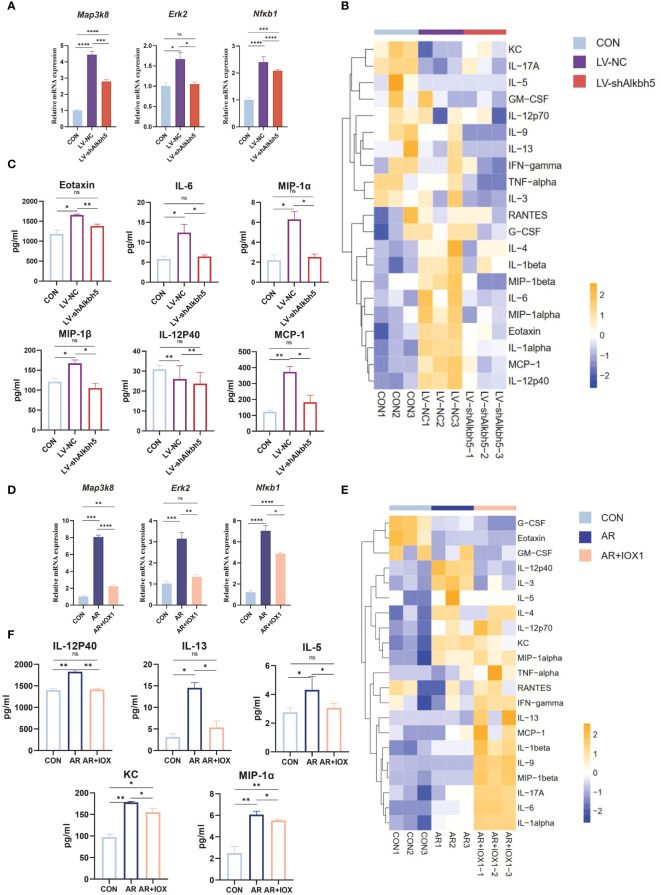
*Alkbh5* knockdown in the nasal mucosa attenuates MAPK pathway upregulation and decreases the inflammatory response. **(A)** Relative mRNA expression levels of *Map3k8, Erk2, and Nfκb1* in nasal mucosa from mice of CON, AR+LV-NC and AR+LVsh*Alkbh5* groups(n=3). **(B)** Heatmap showing the cytokines in serum from CON, AR+LV-NC and AR+LVsh*Alkbh5* groups(n=3). **(C)** Quantification of total cytokine levels of eotaxin, IL-6, IL-12P40, MIP-1α, MIP-1β, and MCP-1 in serum from CON, AR+LV-NC and AR+LVsh*Alkbh5* groups(n=3). **(D)** Relative mRNA expression levels of *Map3k8, Erk2, and Nfκb1* in nasal mucosa from mice of CON, AR and AR+IOX1 groups(n=3). **(E)** Heatmap showing the cytokines in serum from CON, AR and AR+IOX1 groups(n=3). **(F)** Quantification of total cytokine levels of IL-12P40, IL-13, IL-5, KC, and MIP-1α in serum from CON, AR and AR+IOX1 groups(n=3). Data are expressed as mean ± SEMs. *p<0.05, **p<0.01, *** *p* < 0.001 or ****p<0.0001, ns not significant.

**Figure 9:**
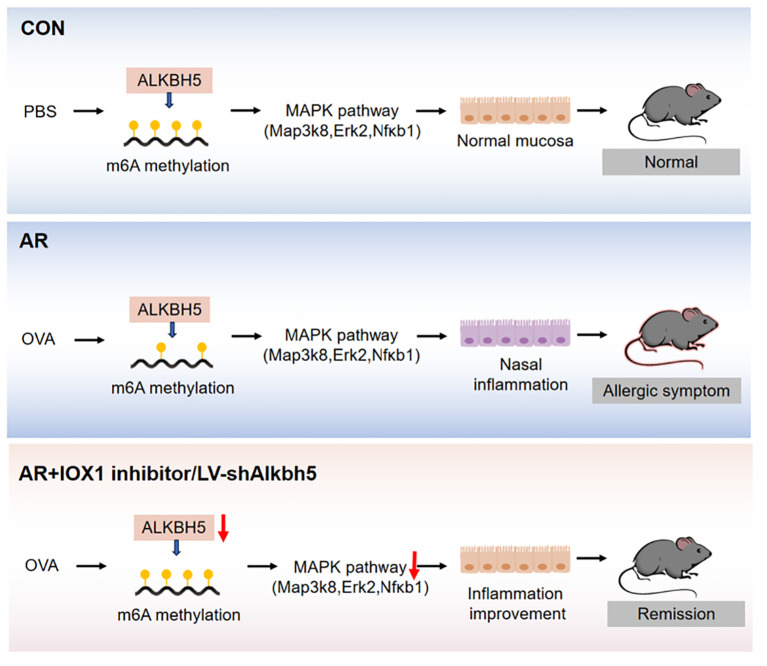
Graphical summary of this study.

*Map3k8, Erk2, and Nfκb1* mRNA levels were increased in the nasal mucosa of AR mice but their m6A levels were decreased. The MAPK pathway may therefore be a target for treating the AR-nasal mucosa-related inflammatory response. First, we investigated whether *Alkbh5* plays a regulatory role in the expression of MAPK genes. Knockdown of *Alkbh5* decreased the mRNA levels of *Map3k8, Erk2, and Nfκb1* (**Figure 8A**). Similar results were obtained using an ALKBH5 inhibitor (**Figure 8D**). This indicates that *Alkbh5* affects the expression of *Map3k8, Erk2, and Nfκb1* in the MAPK pathway. Besides, our results showed that the phosphorylation level of NF-κB (p65) in nasal mucosa of LV-NC group was significantly higher than that of CON group (P=0.0227). The phosphorylation level of NF-κB (p65) in nasal mucosa of LV-shAlkbh5 group was lower than that of LV-NC group(p-NF-κB/NF-κB mean ± SEM:0.5339 ± 0.02353 vs 0.7782 ± 0.09363), although the difference was not statistically significant(P=0.0646) (**Supplementary Figure S7**). Next, we detected expression of 23 mouse cytokines in the nasal mucosa after knockdown of *Alkbh5*. The expression levels of cytokines, such as Eotaxin, IL-6, IL-12p40, MIP-1α, MIP-1β and MCP-1 (**Figures 8B, C**; **Supplementary Table S14**), were altered after knockdown of *Alkbh5*. We also detected altered expression of 23 cytokines after treatment with the ALKBH5 inhibitor, IOX1. Interestingly, the expression of cytokines, such as IL-12p40, IL-13, IL-5, KC and MIP-1α (**Figures 8E, F**; **Supplementary Table S14**), were also altered. These data indicate that ALKBH5-targeted therapy might be effective for treatment of the AR-nasal mucosa inflammatory response (**Figure 9**).

## 4 Discussion

It is well known that m6A RNA methylation plays an important role in immune and inflammatory responses. This study examined the dynamics of m6A methylation in the nasal mucosa through analysis of AR model mice. We found that ALKBH5 positively correlated with m6A modification and nasal mucosa inflammation by regulating the MAPK signaling pathway. In the present study, antigen-induced AR mice were generated by intraperitoneal injection of OVA prior to intranasal instillation. Strong systemic and local allergic responses were revealed by a dramatic increase in OVA-specific IgE, which was consistent with previous studies showing strong immune responses in systemically sensitized AR animals (56–59). We also showed that upper-airway OVA challenge with prior systemic antigen sensitization induced a profound inflammation of the nasal mucosa, characterized by an increased level of inflammatory cells and goblet cells (**Figure 1**). Allergic nasal symptoms of nose scratching and sneezing were also observed. Overall, these results demonstrated the effectiveness of our model.

Although m6A methylation is the most common RNA post-transcriptional modification, which regulates a wide variety of inflammatory diseases (60, 61), the importance of this epigenetic modification in AR is only just beginning to be appreciated. We found prominent m6A modifications in both normal and OVA-induced nasal mucosa. The two groups have approximately the same distribution of the m6A peak characteristics, such as peak frequency and width within mRNA transcripts, and the same distribution of m6A peaks in genic regions. Nevertheless, there were large numbers of unique m6A peaks and methylated mRNAs in AR. Most of the m6A peaks were distributed in 3′-UTRs and promoter regions, which was not consistent with the previously reported peak distribution trend in mouse and human tumor tissues. We considered that such inconsistencies may be related to species, tissue, and condition differences. Our results demonstrated that m6A methylation might have a role in AR. In AR mice, we found that different methylated genes were more strongly correlated with inflammation and immune processes compared with control mice. m6A methylation affects mRNA stability and degradation and relies on the binding of m6A-related RNA-binding proteins to regulate mRNA expression (62). We then analyzed the differentially expressed genes and differentially methylated peaks and noted that many differentially expressed genes with differential m6A peaks in AR are closely related to immune function, such as immune receptor activity and Th17 differentiation. Th17 cells comprise a distinct lineage of proinflammatory T helper cells that are major contributors to allergic responses, especially allergic airway inflammation (63, 64). In addition to the MAPK signaling pathway, MAP kinase phosphatase activity was also significantly enriched.

The MAPK/ERK signaling pathway has been extensively studied in the context of tumor diseases (65, 66), and several lines of evidence indicate that overexpression and activation of ERK/MAPK plays an important role in the progression of allergic inflammation (67–70). Ginsenoside Rh2 inhibits mast cell-induced allergic inflammation, which might be mediated by MAPK signaling pathways (71). Another study indicates that spilanthol may protect against atopic dermatitis skin lesions through inhibition of MAPK signaling to block allergic inflammation (72). AR may involve anti-inflammatory and immunosuppressive effects that may involve MAPK, HIF1, and JAK-STAT pathways. In our study, MAPK/ERK pathway activity was significantly enhanced in AR at transcriptional levels but the m6A methylation was downregulated (**Figures 5E, F**). This corroborates previous studies reporting that m6A methylation decreases gene expression (73, 74). In the OVA-induced AR mouse model, the expression of Map3k8 and NF-κB was increased in the nasal mucosa. This indicates that MAPK may play a pivotal role in the pathology of AR. NF-κB and p38 MAPK are transcription factors activated by IL-25. Up-regulation and activation of NF-κB promotes Th2 cell proliferation and cytokine production, while numerous studies show the degree of allergic inflammation is mediated by NF-κB and p38 MAPK pathways. Our results provided a possible role for m6A and its downstream transcription factors in the MAPK signaling pathway in AR.

We determined that the mRNA and protein levels of ALKBH5 were higher in the nasal mucosa of AR mice compared with those in control mice (**Figure 6**). ALKBH5, an important modifier of m6A methylation, has been reported in other immune diseases (75–77). For example, ALKBH5 is highly expressed in lung adenocarcinoma cells under intermittent hypoxia, and knockdown of ALKBH5 inhibits the proliferation and invasion of lung adenocarcinoma cells by reducing the m6A level of *Foxm1 (* 78). Nonetheless, recent reports show that ALKBH5 plays an inhibitory role in bladder cancer (79, 80). Moreover, ALKBH5 knockdown inhibits cell viability, induces apoptosis, and decreases inflammatory cytokine production by LPS-treated HK-2 cells (81). It is speculated that ALKBH5 plays a role in regulating different target genes or m6A modifications in different regions of the same gene via different reading proteins.

More than a dozen completed clinical trials have used lentiviral vectors for treating a range of diseases, including metabolic disorders, cancers, immune disorders, and rare congenital diseases (80). Furthermore, lentiviruses have been administered intranasally in many successful trials (82). Intranasal corticosteroids are primary treatments for patients with AR and have favorable efficacy and safety. Administration of lentiviruses overexpressing Notch2 (46), miR-224–5p (83) and miR-135a by nasal drip can significantly reduce the systemic inflammatory response in mice with AR. In our study, the behaviors of sneezing and nose-scratching observed during transient knockdown of *Allkbh5* in AR mice confirmed that dysregulation of *Allkbh5* affects nasal functions (**Figure 7**). AR symptoms are concentrated in the nasal tissues and the width of the nasal cavity, which is influenced by inflammatory cells, plays a central role in AR. Hypertrophic and metaplastic changes of goblet cells associated with mucous hypersecretion are common characteristics of airway inflammation. Mucous hypersecretion is an important clinical observation in allergic inflammation of the respiratory epithelium, such as in bronchial asthma, because an excessive production of mucus causes plugging in the lower airways that may lead to airway obstruction and progressive respiratory insufficiency. The hypersecretory mucus production by goblet cells in AR appears to be a major contributor to the severity of the disease.

Serum levels of IL-6, MIP-1α, MIP-1β, and IL-12p40 have been used to predict the development of inflammatory disease (84–87) and are decreased in AR mice after lentivirus nasal drop administration. Eotaxin is a small protein that is a potent chemoattractant for eosinophils (88). Knockout of *Alkbh5* in mice led to lower eotaxin levels. Whereas, as inhibition and lentiviral knock-down of ALKBH5 reveals different results in expression levels of cytokines, possibly due to the lower specificity of IOX1 inhibitors, which inhibited the activity of ALKBH5 enzyme while also affecting the expression level of some other key enzymes after injection intervention. Since that previous studies have shown that IOX1 can significantly inhibit the expression of ALKBH5. However, IOX1 is a broad-spectrum inhibitor that can also inhibit enzymes including Jumonji C(JmjC) demethylases. The JmjC domain can demethylate histones by an oxidative mechanism requiring Fe(II) and alpha-ketoglutarate (αKG) as cofactors. For these differences, further exploration is needed. Since ALKBH5 rely on cofactors 2OG and Fe2+ for their m6A demethylation activity, IOX1 decreased the activity of ALKBH5 by inhibiting the activity of 2OG oxygenases effectively, and this property of IOX1 is beneficial to explore new drugs for AR treatment in the future. We speculate that *Alkbh5* regulates development of AR through its effects on inflammation. MAPKs, such as ERK, p38, and NF-κB, are closely involved in the synthesis of inflammation mediators. Therefore, inhibitors targeting MAPKs have been developed to reduce inflammation. We found that knockdown of ALKBH5 was accompanied by decreased levels of *Map3k8, Erk2, and Nfκb1* mRNAs. Although the effect of ALKBH5 on MAPK signaling pathway needs to be confirmed by further studies, targeting ALKBH5 may help prevent nasal inflammation in AR (**Figures 8**, **9**).

Our study provides insight into m6A changes occurring in nasal mucosa of AR. These results indicate that RNA methylation modifications in the nasal mucosa of AR affect allergic symptoms through the MAPK pathway. Furthermore, our work demonstrates the critical role of ALKBH5, which can cause aberrant expression of *Map3k8, Erk2, and Nfκb1* at the transcriptional level. These findings indicate that ALKBH5 is a potential therapeutic target for AR via modulation of the MAPK signaling machinery. These results show that understanding the molecular mechanisms of AR-induced nasal mucosa-related allergic symptoms can lead to new treatments for allergy-related diseases.

## 5 Conclusion

Taken together, we mapped the first mouse m6A methylomic landscape in the nasal mucosa tissues following OVA-induced AR and conclude that m6A dysregulation mediated by ALKBH5, is likely to contribute to inflammation of the nasal mucosa via the MAPK signaling pathway, suggesting that ALKBH5 is a potential biomarker for AR treatment. Our results provided a novel direction for understanding the mechanism of m6A methylation affecting allergic inflammation and screening potential therapeutic targets in AR.

## Data availability statement

The datasets presented in this study can be found in online repositories. The names of the repository/repositories and accession number(s) can be found below: https://www.ncbi.nlm.nih.gov/, PRJNA1044931.

## Ethics statement

The animal studies were approved by Capital health development project. The studies were conducted in accordance with the local legislation and institutional requirements. Written informed consent was obtained from the owners for the participation of their animals in this study.

## Author contributions

RW: Conceptualization, Data curation, Methodology, Supervision, Visualization, Writing – original draft, Writing – review & editing. JL: Data curation, Methodology, Writing – review & editing, Investigation, Project administration. QW: Data curation, Methodology, Writing – review & editing. YZ: Writing – review & editing, Data curation, Methodology. YL: Project administration, Writing – review & editing, Supervision, Validation. XZ: Investigation, Project administration, Writing – review & editing, Formal analysis. SW: Investigation, Project administration, Resources, Supervision, Visualization, Writing – review & editing, Conceptualization, Validation. QG: Investigation, Project administration, Writing – review & editing, Funding acquisition, Resources, Supervision, Visualization.
