# Supplementary figures and images for: m6A mRNA methylation-mediated MAPK signaling modulates the nasal mucosa inflammatory response in allergic rhinitis
Source: Front Immunol. 2024 Jul 1;15:1344995. doi: 10.3389/fimmu.2024.1344995 (PMC11246857; doi:10.3389/fimmu.2024.1344995)

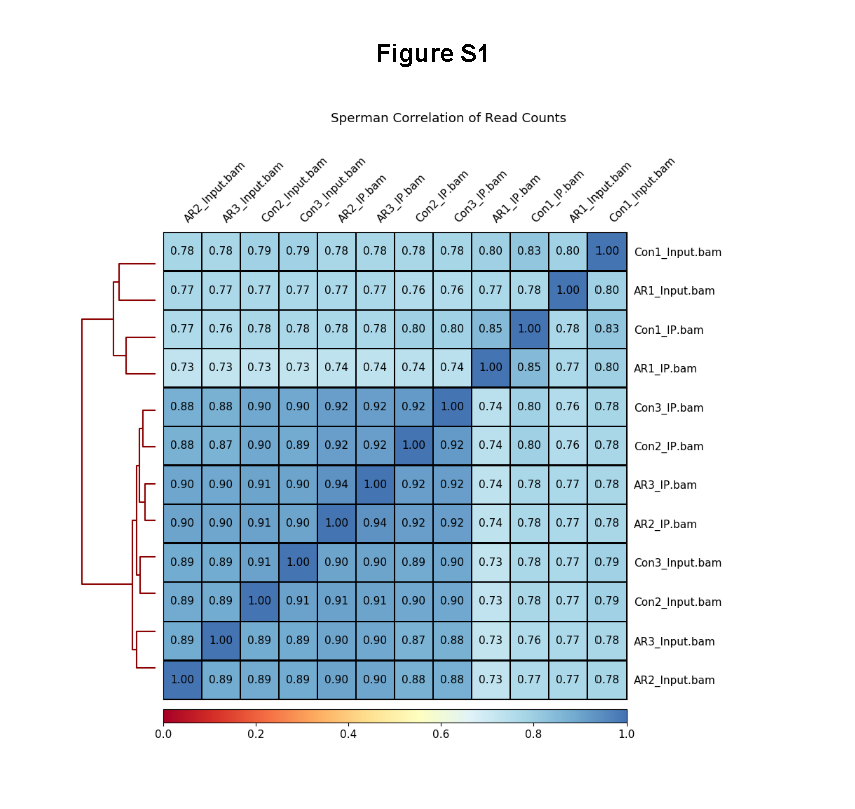

Supplement: Supplementary file 16 [file Image_1.tif]

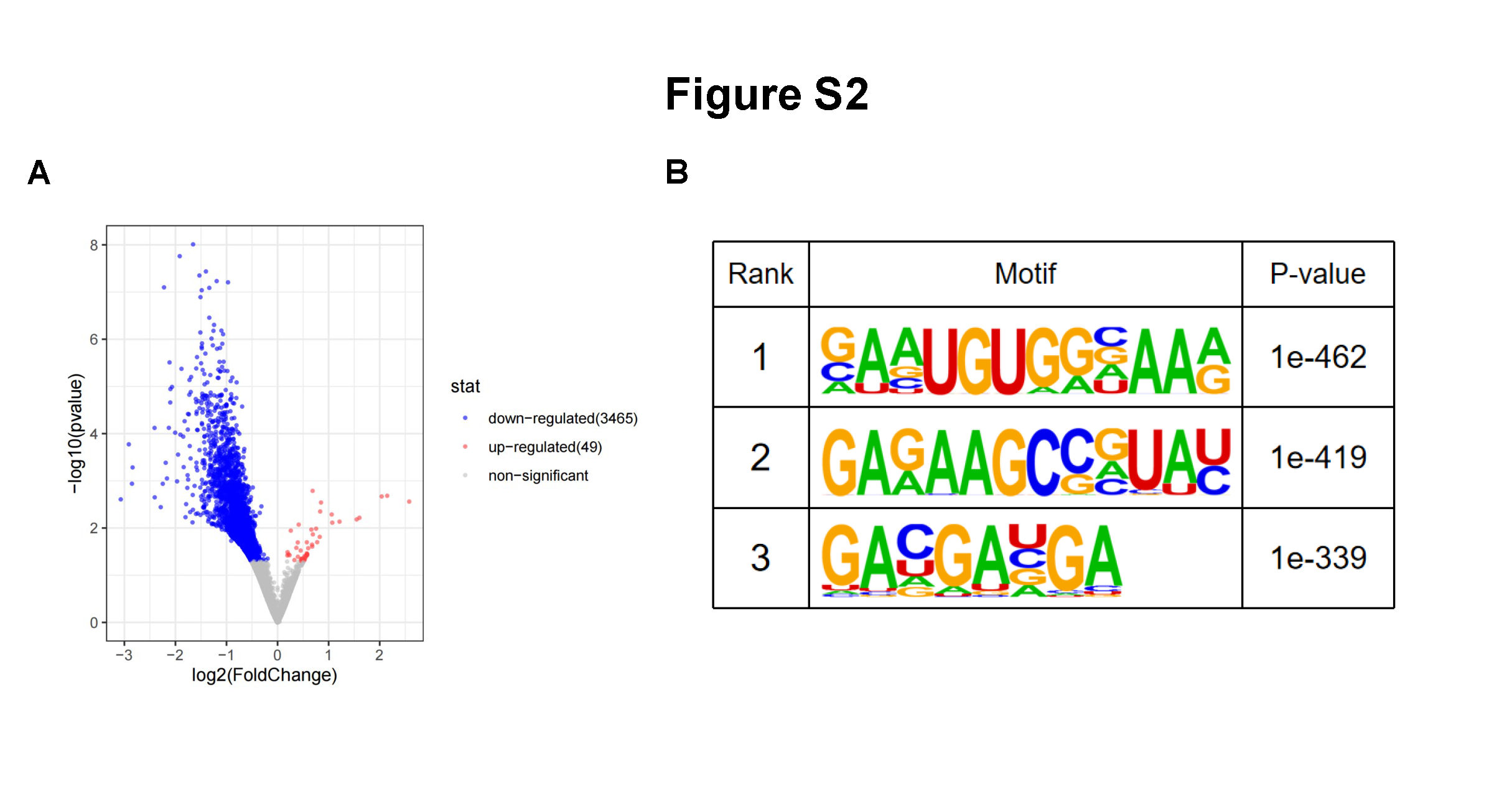

Supplement: Supplementary file 17 [file Image_2.tif]

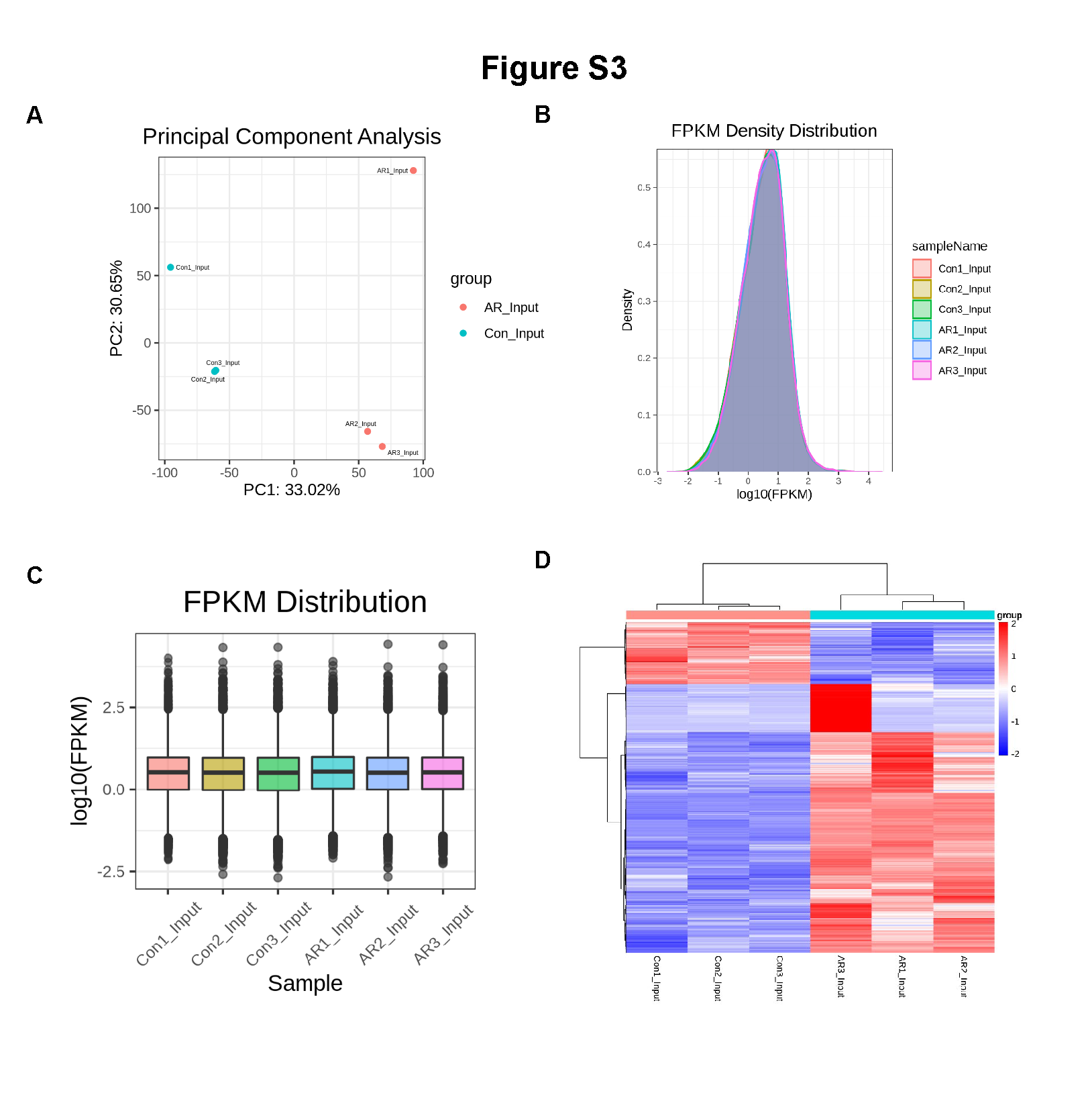

Supplement: Supplementary file 18 [file Image_3.tif]

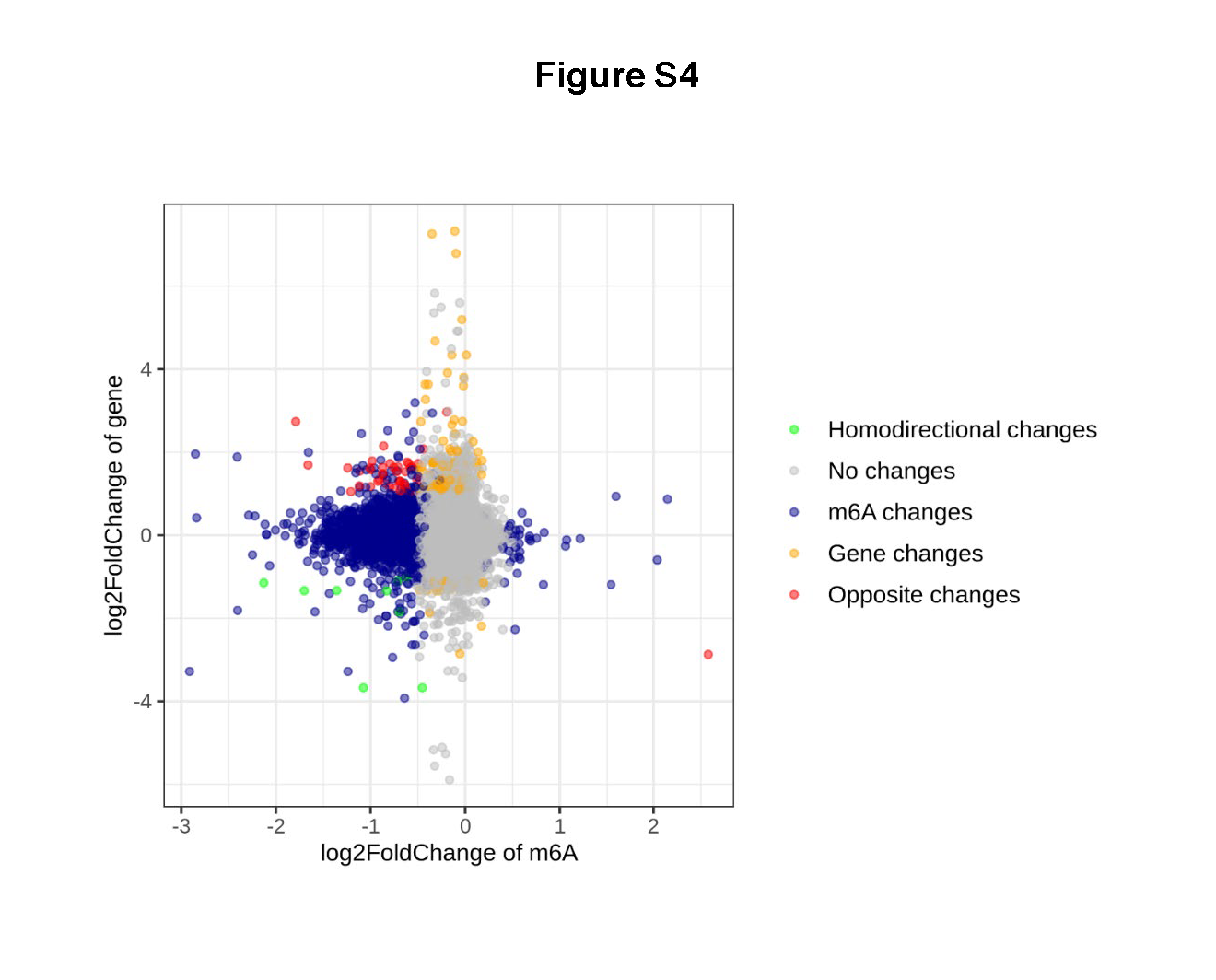

Supplement: Supplementary file 19 [file Image_4.tif]

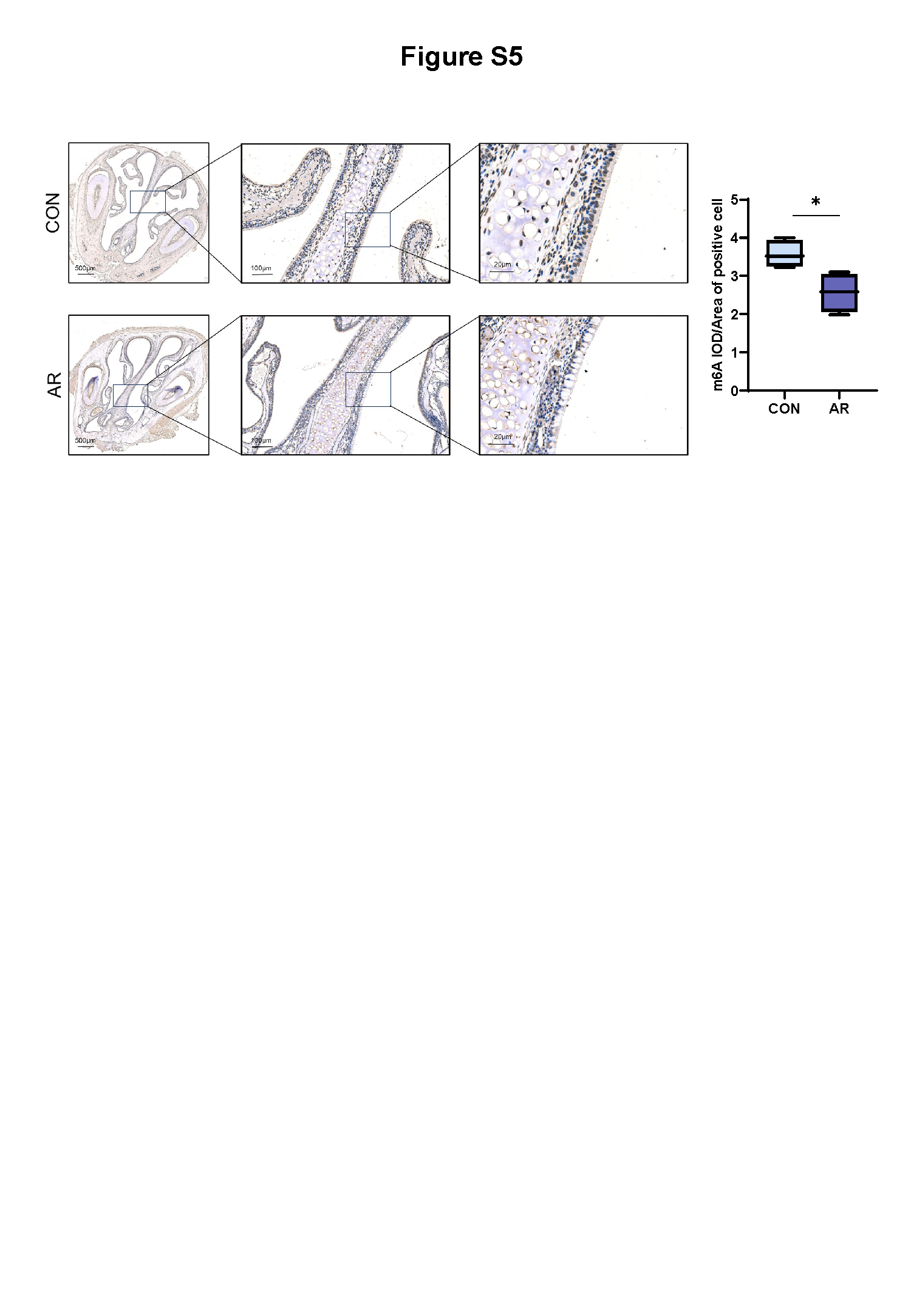

Supplement: Supplementary file 20 [file Image_5.tif]

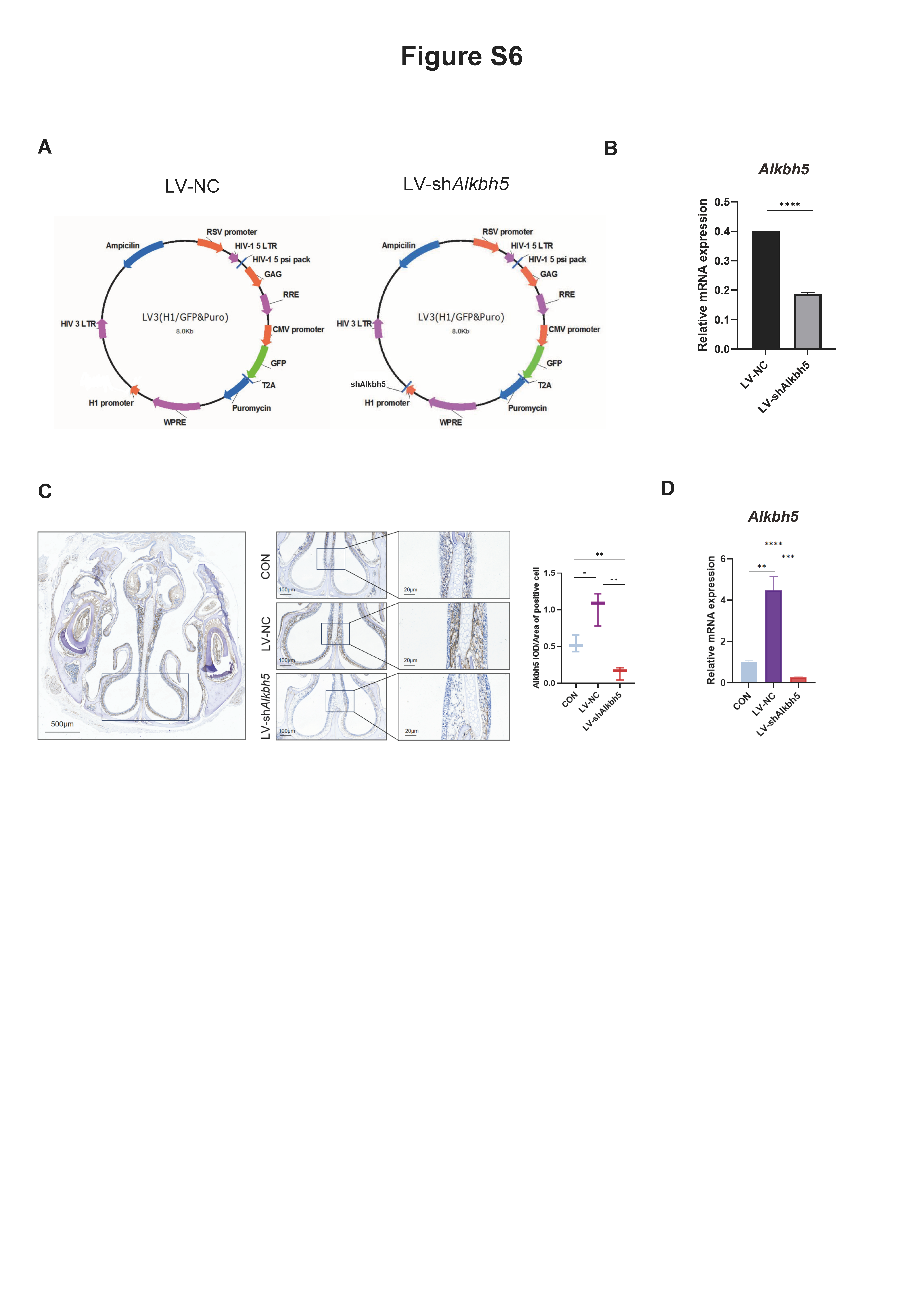

Supplement: Supplementary file 21 [file Image_6.tif]
